# The effect of prone and supine treatment positions for the pre-operative treatment of rectal cancer on organ-at-risk sparing and setup reproducibility using volumetric modulated arc therapy

**DOI:** 10.1186/s13014-017-0918-5

**Published:** 2017-12-05

**Authors:** Anthony Kim, Aliaksandr Karotki, Joe Presutti, Glen Gonzales, Shun Wong, William Chu

**Affiliations:** 1 Sunnybrook Health Sciences Centre/Odette Cancer Centre, 2075 Bayview Avenue, Toronto, ON M4N 3M5 Canada; 2 Department of Radiation Oncology, University of Toronto, Toronto, ON Canada

**Keywords:** Rectal cancer, VMAT, CBCT, Prone, Supine

## Abstract

**Background and purpose:**

To compare organ-at-risk doses and setup reproducibility using the prone and supine orientations in volumetric modulated arc therapy (VMAT) for rectal cancer.

**Materials and methods:**

Seventeen consecutive rectal cancer patients undergoing preoperative radiation were selected and setup in either the prone (*N* = 8) or supine (*N* = 9) position. All patients were treated using posteriorly-applied VMAT. Bladder and small bowel dose and cone beam CT (CBCT) reproducibility metrics were retrospectively collected.

**Results:**

Dose metrics for bladder and small bowel did not show significant differences between the prone and supine orientations. The prone data had a trend for smaller irradiated volumes than supine for the small bowel at lower doses—V20 (prone: 135 ± 99 cm^3^; supine: 201 ± 162 cm^3^) and V30 (prone: 78 ± 71 cm^3^; supine: 105 ± 106 cm^3^). At higher doses, the trend reversed as exemplified by the small bowel V50.4 (prone: 20 ± 28 cm^3^; supine: 10 ± 14 cm^3^). CBCT data showed that rotational errors in pitch and roll were significantly larger for the prone vs. supine orientation (pitch: 2.0° ± 1.3° vs. 0.8° ± 1.1° *p* < 0.001; roll: 1.0° ± 0.9° vs. 0.3° ± 0.5°, *p* < 0.001).

**Conclusions:**

Bladder and small bowel doses were not significantly different when comparing VMAT plans developed for the prone and supine orientations. The supine orientation demonstrated improved setup reproducibility.

## Introduction

Globally and every year, over 1.4 million people are diagnosed with colorectal cancer [1, 2]. In 2015, it was the fourth most common malignancy (49.9 cases per 100,000) in Canada [3].

Preoperative radiotherapy or chemoradiation followed by total mesorectal excision (TME) is currently the standard of care for patients with locally advanced rectal cancer [4, 5]. The small bowel is the most relevant organ-at-risk (OAR) nearby typical rectal cancer target volumes. A standard prescription for preoperative radiotherapy is 50.4 Gy in 28 fractions, which may be high enough to elicit small bowel complications [6]. In an effort to avoid small bowel toxicities, many centers treat patients in the prone position propped up on belly boards where the abdomen falls through a hole in the board to allow the small bowel to drop anteriorly away from the target volume [7].

Volumetric modulated arc therapy (VMAT) is increasingly used to deliver radiation treatment for a variety of sites, including rectal cancer [8]. VMAT allows for rapid treatment delivery and highly conformal dose distributions compared to 3D conformal radiation therapy (3DCRT) or intensity modulated radiation therapy (IMRT). Previously, our center treated rectal patients using a 3-beam 3DCRT technique. Recently, we adopted VMAT for rectal cancer treatment, employing a ~180° posterior arc delivering conformal dose distribution whilst avoiding beam entry anteriorly through the small bowel and bladder. Treating with VMAT has necessitated the use of cone-beam CT (CBCT) in-room image guidance for patient setup verification to improve accuracy of dose delivery.

The prone orientation for rectal cancer patients is known to cause patient discomfort, especially for patients with a stoma. The combination of a belly board with the prone position is also known for setup errors [9]. Thus the supine treatment position is an attractive alternative. With the implementation of VMAT and CBCT, we assessed if the supine position can be safely used to treat rectal cancer. Our objectives were *i*) to compare small bowel and bladder doses and *ii*) to compare the setup reproducibility using CBCT for the prone and supine orientations.

## Materials and methods

### Patient selection

This study was conducted as a retrospective review approved by the institutional research ethics board. Prior to the study, rectal cancer patients undergoing preoperative radiation were treated either prone or supine as per the radiation oncologists using the VMAT technique. Seventeen consecutive patients (8 prone and 9 supine setup) were selected for this review. The only exclusion criteria were non-standard VMAT beam arrangements and prostheses within the axial treatment planes.

### Simulation and treatment planning

Prone patients were simulated and treated on a carbon fiber belly board (Model# 125012 from Civco Medical Solutions, Coralville, Iowa, USA); supine patients were simulated and treated on a flat styrofoam board to reduce posterior skin dose. All patients had the GTV, CTV, PTV, and pelvic vessels contoured as per our clinical protocol. The GTV represented the rectal cancer based on the diagnostic MRI. The CTV included an expansion around the GTV, the entire mesorectum, and 1.5 cm expansion around the internal iliac vessels up to their bifurcation. The PTV was generated by forming a 1 cm isotropic margin about the CTV. Patients were simulated with a comfortably full bladder. No intravenous contrast was used.

**Fig. 1:**
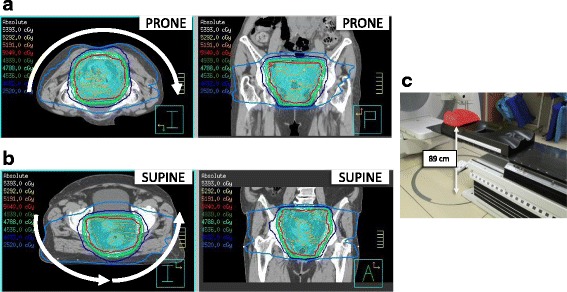
Prone (**a**) and supine (**b**) VMAT rectum treatment plans. Axial and coronal slices are shown. The arrows indicate the VMAT arcs, which were always posteriorly applied. The supine VMAT plan necessarily has two arcs due to the Elekta linacs’ inability to over-travel past gantry angle 180°. **c** A carbon fiber belly board used for prone setup of rectal cancer patients. The belly board is setup on a Hexapod couch in this image (not used for rotational correction for rectal cancer patients at our center). The top of the belly board is 89 cm above the floor at the lowest position

Clinical treatment plans were generated in the Pinnacle^3^ v9.8 treatment planning system (TPS), for delivery on either the Elekta (Crawley, UK) Synergy MLCi or Synergy Agility treatment delivery platforms. The treatment beam geometry was a 6 MV ~180° posterior VMAT arc for all patients regardless of setup orientation (Fig. 1). We employ a treatment couch model in our TPS that is a 1 cm water equivalent structure placed at the level of the couch top—this is particularly important for the supine treatment plans as the couch attenuates the 6 MV beam by approximately 2%. Anterior dose sculptors were added to steer dose away from the small bowel and bladder. Our standard preoperative rectal cancer prescription is 50.4 Gy in 28 fractions. One supine setup patient was prescribed a dose of 25 Gy in 5 fractions preoperatively, and another prone setup patient was treated definitively with a dose of 40 Gy in 15 fractions (for the dosimetric analysis of small bowel and bladder, the prescription dose for these patient plans were normalized to 50.4 Gy for consistency). Planners strove to meet a target coverage of V50.4 > 99% for the CTV and V47.9 > 99% for the PTV, with the 105% hot spot limited to <1% of the PTV volume, which was achieved for every patient in the study.

Individual small bowel loops were contoured within 2 cm of the PTV superiorly by one of two staff radiation oncologists (WC and SW) and reviewed by the other. The bladder was contoured by a dosimetrist and reviewed by the two radiation oncologists. These contours were generated retrospectively for this study and were not involved in the clinical plan optimization. The small bowel mean dose, V50.4, V45, V30, V20, and V15 and bladder mean dose, V40, and V30 were collected retrospectively. Student’s *t*-tests using Matlab (Natick, MA, USA) were employed to parse out the statistically significant differences in the DVH metrics between prone and supine orientations.

### CBCT and reproducibility metrics

To quantify the setup reproducibility of the patients, for each patient one CBCT per week was retrieved from archive and loaded into the Pinnacle TPS. The single exception was one of the patients that had a prescription of 25 in 5 fractions—in this case every CBCT was analyzed. The rotational error was quantified using the Image Fusion module in Pinnacle in the pitch, yaw, and roll directions. In this paper, the pitch direction refers to rotation about the left-right axis, and is related to the tilt of the pelvis; the yaw direction refers to rotation about the anterior-posterior axis, and is related to superior-inferior straightening of the patient; the roll direction refers to rotation about the cranial caudal axis (i.e. in the “barrel roll” direction). Statistical significance between the prone and supine study arms was tested using the Student’s *t*-tests on each of the rotational direction data sets.

## Results

### Patient, tumour, and treatment characteristics

Patient, tumour and treatment characteristics were similar for the prone and supine treatment groups as summarized in Table 1. All 17 patients in this study were treated with curative intent for clinical ≥T3 or node positive disease.

**Table 1:** Patient, tumor, and treatment characteristics

Characteristic	Prone *n* = 8 (%)	Supine *n* = 9 (%)
Median age, yrs. (range)	65 (40–86)	78 (47–86)
Sex		
M F	4 (50) 4 (50)	5 (56) 4 (44)
Tumour status^a^		
T1–2 T3 T4	0 (0) 6 (75) 2 (25)	0 (0) 8 (89) 1 (11)
Node status		
Positive Negative	4 (50) 4 (50)	6 (67) 3 (33)
Radiation Preoperative Definitive RT dose	7 (88) 1 (12) 50.4 Gy in 28 (*n* = 7) 40 Gy in 15 (*n* = 1)	9 (100) 0 (0) 50.4 Gy in 28 (*n* = 8) 25 Gy in 5 (*n* = 1)
Concurrent chemotherapy		
Capecitabine None	7 1	8 1

^a^Clinical stage based on diagnostic MRI

### Bladder and small bowel DVH metrics

**Fig. 2:**
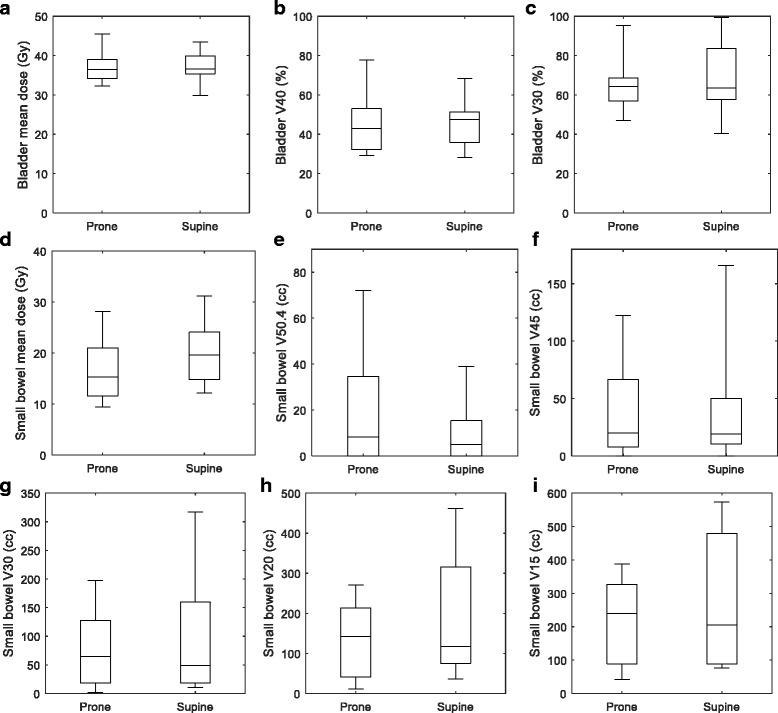
Bladder **a** mean dose, **b** V40, and **c** V30 box-whisker plots for prone and supine orientations. Small bowel **d** mean dose, **e** V50.4, **f** V45, **g** V30, **h** V20, and **i** V15 box-whisker plots. The first and third quartiles are indicated by the ends of the box, with the line in the middle indicating the median. The “whiskers” display the maximum and minimum of the data. No outliers were considered in these plots

All bladder DVH metrics were not significantly different between prone and supine study arms. Table 2 and the box-whisker plots of Fig. 2a, b, and c show that the data for prone vs supine were comparable.

**Table 2:** Bladder and small bowel DVH metrics for the prone and supine orientations. (average ± standard deviation)

DVH Metric	Prone	Supine	*p* value
Bladder mean dose (Gy)	37.1 ± 4.2	37.3 ± 4.1	0.93
Bladder V40 (%)	45.4 ± 16.3	46.0 ± 12.6	0.93
Bladder V30 (%)	65.2 ± 14.4	68.8 ± 18.5	0.67
Small bowel mean dose (Gy)	16.7 ± 6.4	19.8 ± 6.3	0.32
Small bowel V50.4 (cc)	19.7 ± 27.8	10.3 ± 13.9	0.38
Small bowel V45 (cc)	38.9 ± 44.7	37.5 ± 51.6	0.95
Small bowel V30 (cc)	77.6 ± 70.6	104.9 ± 106.1	0.55
Small bowel V20 (cc)	134.5 ± 99.1	201.2 ± 161.7	0.33
Small bowel V15 (cc)	217.2 ± 135.1	260.2 ± 198.7	0.61

The average small bowel volumes (average ± standard deviation) contoured by the radiation oncologists were similar between prone (521 ± 226 cm^3^) and supine patients (489 ± 211 cm^3^) (*p* = 0.8). Table 2 also shows the DVH metrics for small bowel, with the data displayed in more detail in the box-whisker plots of Fig. 2d, e, f, g, h, and i. The average values of the small bowel V50.4 and V45 were lower for the supine compared to the prone patients; however, at the low-intermediate dose levels the trend reversed, with the V30, V20, and V15 being lower for the prone patients. Specifically, the V15 was 260 ± 199 cm^3^, and V50.4 10 ± 14 cm^3^ for patients treated in the supine position; in the prone position, V15 was 217 ± 135 cm^3^ and the V50.4 20 ± 28 cm^3^. The mean dose was slightly lower on average for the prone patients. All DVH metrics for the small bowel did not exhibit statistically significant differences between prone and supine patients.

### CBCT evaluation of rotational errors

**Fig. 3:**
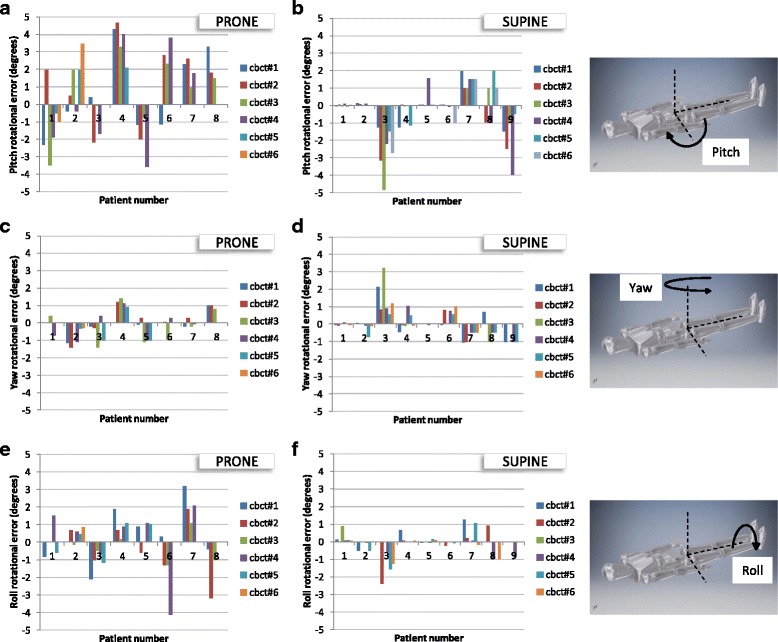
Rotational errors between planning CT and treatment CBCT; **a** pitch/prone, **b** pitch/supine, **c** yaw/prone, **d** yaw/supine, **e** roll/prone, **f** roll/supine

Figure 3a and b show the pitch rotational errors for the prone and supine study arms. Based on these absolute values, the mean pitch error was 2.5× greater in the prone compared to supine orientation (1.97 ± 1.28 vs 0.80 ± 1.08; *p* < 0.001; Table 3).

**Table 3:** Mean pitch, yaw and roll rotational errors as determined from CBCT matches with the planning CT

Orientation	Pitch error (deg)	Yaw error (deg)	Roll error (deg)
Prone	1.97 ± 1.28	0.57 ± 0.49	1.04 ± 0.94
Supine	0.80 ± 1.08	0.46 ± 0.59	0.35 ± 0.53
*p value*	<0.001	0.35	<0.001

The rotational data for a given direction (pitch, yaw, or roll) and patient orientation (prone or supine) were taken as the absolute value and then averaged together (i.e. the magnitudes of the rotational errors are averaged together). The ± appendages denote the standard deviation

The yaw rotational errors were similar in the two study arms as displayed in Fig. 3c and d and quantified in Table 3. The *t*-test demonstrated no statistically significant difference between the prone and supine study arms.

The roll rotational errors (Fig. 3e and f) were 3× larger in the prone compared to supine orientation (1.04 ± 0.94 vs 0.35 ± 0.53, p < 0.001; Table 3).

## Discussion

To date, this is the first study to report dosimetric data for OARs and setup reproducibility for prone and supine orientations in the context of VMAT treatment for rectal cancer. This study was driven by our clinical need to determine if treating patients in the supine position is preferable to the prone position in light of our adoption of VMAT and CBCT-mediated image-guided RT for rectal cancer.

Use of the belly board and the prone position for rectal cancer was born of the need to reduce small bowel toxicity. This was prominent in the era of 3DCRT when prone positioning on a belly board and/or distending the bladder were used to move the small bowel away from the treatment volume [10]. The aim of reducing bowel toxicity comes with a trade-off of reduced patient reproducibility and stability. A previous study demonstrated that using a belly board increased anterio-posterior reproducibility displacements compared with no belly board in the prone position [9]. Positional changes would be expected when considering the smooth, curved structure of the belly board and differences in patient habitus. Our radiation therapists had reported that patients’ abdomens fall variably into the belly board cavity at every fraction, thus, we anticipated that the pitch direction would be more variable for the prone orientation. Our CBCT data confirms that a belly board introduces set-up errors in the pitch and roll rotational directions. We did not observe any differences in the yaw direction, as this is arguably the direction the therapists have the most control over with the aid of a superior straightening tattoo.

There are safety considerations that would warrant moving to the supine position. Figure 1c shows how high a patient needs to vault in order to mount a belly board, a potentially hazardous situation for elderly or frail patients. During the course of this work, there was a non-study patient that fell off one of the prone belly boards, triggering an incident investigation at our institution. The supine setup is more comfortable for patients and convenient for therapists on the treatment units [11].

Various studies have compared prone and supine orientations for rectal cancer treatment and reported the dosimetric impact on the small bowel. Dryzmala et al. have simulated rectal cancer patients in both the prone and supine positions and created clinical-grade 3DCRT plans for both orientations [12]. Nijkamp et al. tested two prone belly boards and the supine position on volunteers scanned in an MRI and planned treatments using 7-beam IMRT [13]. The treatment modalities discussed above are likely not optimal for avoiding small bowel dose, where three-field techniques are limited in their ability to sculpt dose away from anterior structures, and isotropically placed IMRT beams (e.g. 7-field techniques) place several entry beams through the small bowel on the way to the rectal target. The results from both studies broadly agree with each other in that the belly board significantly reduced the volume of irradiated small bowel in the low-intermediate dose range (Dryzmala: 5–15 Gy; Nijkamp: <35 Gy), while no significant differences were seen in the high dose range (Dryzmala: 20–45 Gy; Nijkamp: 50 Gy). Additional data from Froseth et al. demonstrated that at dose levels from 5 to 45 Gy (tested at 5 Gy increments) there was no statistically significant difference between irradiated volumes when comparing a prone setup study arm (*N* = 40) with a supine study arm (*N* = 43) using a 3DCRT planning strategy [14]. Our data also showed a similar trend to the Dryzmala and Nijkamp studies with a smaller volume of irradiated small bowel in the low dose range with prone versus supine patients. At the high dose range (V45 and V50.4) the volume of irradiated small bowel was lower in supine patients. It should be noted that none of our OAR dosimetric data showed statistically significant differences between the prone and supine positions. One possible reason for the lack of differences is the use of a posteriorly-applied VMAT half arc. Highly conformal dose distributions are possible (Fig. 1) with nearly none of the beam angles entering through the small bowel and bladder on the way to the target. Joye et al. produced a study using VMAT full arcs comparing the full bowel, small bowel, and bowel bag DVH metrics, and found statistically significant differences in the irradiated volumes for V15 and lower doses [15]; this suggests that our technique of using a VMAT posterior half arc may have an additional bowel sparing effect compared with a full arc geometry.

Acute small bowel toxicity is a concern for rectal cancer patients receiving preoperative radiotherapy. Baglan et al. (2002) demonstrated a strong relationship between the volume of small bowel receiving at least 15 Gy and the risk of toxicity [16]. Grade 0–1 toxicity was seen after irradiation with a V15 of less than 150 cm^3^ (grade 0–1 in 90%, grade 2 in 10% of the patients), and 70% of the patients with a V15 of more than 300 cm^3^ experienced grade 3 toxicity. Roeske et al. (2003) demonstrated an increased risk of acute grade 3 toxicity with a V45 greater than 200cm^3^ [17]. VMAT is an advanced method to spare OARs for any treatment site, particularly compared to 3DCRT techniques. The general consensus is that there are dosimetric benefits to VMAT with toxicity levels that are lower than for 3DCRT [18, 19]. In our study, the small bowel V15 was greater than 300cm^3^ for some patients treated in the prone and supine positions, while V45 was consistently less than 200cm^3^. Review of the patient treatment records did not reveal any grade 3 or greater acute GI toxicity in our study. However, a clear limitation of our study is the lack of prospective toxicity assessment. Nonetheless, based on the low volume of irradiated small bowel we expected minimal toxicity. Furthermore, a study from the University Medical Center at Göttingen, Germany demonstrated that VMAT substantially reduced high-grade acute toxicity compared with 3DCRT (5% versus 20% incidence), as well as late toxicity (6% versus 22% incidence) [8].

In addition to the prone orientation being less reproducible than supine, the belly board may unintentionally push the bowel into the treatment volume. In consideration of this, it is a reasonable supposition that the delivered dose distribution is markedly different than the plan dosimetry for many prone patients due to inaccuracies in the patient setup. The rotational setup error can also introduce considerable targeting error. A rudimentary calculation can demonstrate this effect. Several CBCTs showed > ± 4°rotational error in the pitch direction (Fig. 3). Rectal cancer PTV volumes can be 20 cm long in the superior-inferior direction. With the beam isocenter in the middle of the PTV, and a 4° error in pitch, this can result in the superior and inferior ends of the PTV being displaced by 7 mm, which would be deleterious for target coverage and (if rotated in a disadvantageous direction) small bowel sparing.

The results drawn from this study were based on rectal cancer patients undergoing preoperative radiotherapy and may not be applicable to rectal cancer patients undergoing postoperative adjuvant radiotherapy.

## Conclusions

In conclusion, we found non-significant dose data differences in the bladder and small bowel between the prone and supine study arms, and greater rotational set-up errors with the prone position. As such, our results support treating all preoperative rectal cancer patients in the supine position. The improved conformality of VMAT aided by the superior setup imaging of CBCT further enables treatment in the supine position, which is more comfortable, reproducible, and safer.

## Abbreviations

3DCRT: 3D conformal radiation therapy
CBCT: Cone beam computed tomography
CT: Computed tomography
CTV: Clinical target volume
DVH: Dose-volume histogram
GTV: Gross tumor volume
IMRT: Intensity modulated radiation therapy
MRI: Magnetic resonance imaging
MV: Megavoltage
OAR: Organ-at-risk
PTV: Planning target volume
TME: Total mesorectal excision
TPS: Treatment planning system
VMAT: Volumetric modulated arc therapy
